# Asymmetric patterning drives the folding of a tripodal DNA nanotweezer†

**DOI:** 10.1039/d1sc04793k

**Published:** 2021-11-16

**Authors:** Daniel Saliba, Tuan Trinh, Christophe Lachance-Brais, Alexander L. Prinzen, Felix J. Rizzuto, Donatien de Rochambeau, Hanadi F. Sleiman

**Affiliations:** Department of Chemistry, McGill University 801 rue Sherbrooke West Montreal QC H3A 0B8 Canada hanadi.sleiman@mcgill.ca

## Abstract

DNA tweezers have emerged as powerful devices for a wide range of biochemical and sensing applications; however, most DNA tweezers consist of single units activated by DNA recognition, limiting their range of motion and ability to respond to complex stimuli. Herein, we present an extended, tripodal DNA nanotweezer with a small molecule junction. Simultaneous, asymmetric elongation of our molecular core is achieved using polymerase chain reaction (PCR) to produce length- and sequence-specific DNA arms with repeating DNA regions. When rigidified, our DNA tweezer can be addressed with streptavidin-binding ligands. Full control over the number, separation, and location of these ligands enables site-specific streptavidin recognition; all three arms of the DNA nanotweezer wrap around multiple streptavidin units simultaneously. Our approach combines the simplicity of DNA tile arrays with the size regime normally provided by DNA origami, offering an integrated platform for the use of branched DNA scaffolds as structural building blocks, protein sensors, and dynamic, stimuli-responsive materials.

## Abstract 2

An extended, multivalent DNA nanotweezer that undergoes large-scale molecular motion upon protein recognition is presented. Our method based on “printing-elongation-folding” combines the DNA-minimal aspect of DNA tile-based assembly, with complexity of DNA origami.

## Graphical abstract

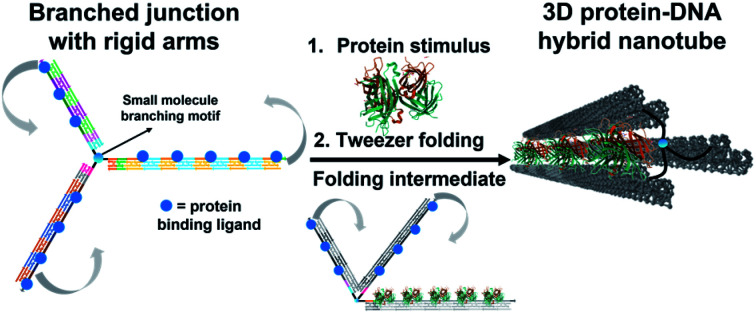

## Introduction

Molecular tweezers are synthetic hosts with open cavities, often composed of multiple branching arms connected by a junction moiety.^1–5^ The rigidity of this hinge is a strong determinant of tweezer properties: rigid spacers result in pre-organized concave binding sites, while more flexible spacers lead to a mechanical pincer-like motion that responds to the spatial and chemical requirements of guest binding.^1,6,7^ DNA tweezers have emerged as powerful nanomechanical devices, with the ability to sense nucleic acids, proteins, and cellular processes, to control enzymatic activity, and measure biological distances.^8–18^ Most DNA tweezers translate molecular recognition (typically another DNA strand) into short-range mechanical motion, resulting in a signal or function.^13,19–27^ Extended DNA tweezers with arms containing multiple binding sites along their lengths would be able to translate multiple guest binding events into large-scale mechanical motion, reminiscent of octopus or squid tentacles around prey. This large-scale motion may find applications in molecular robotics, biosensing schemes with amplification, and cellular probes that interrogate and influence large sections of the cell membrane.^26,28–39^

**Fig. 1:**
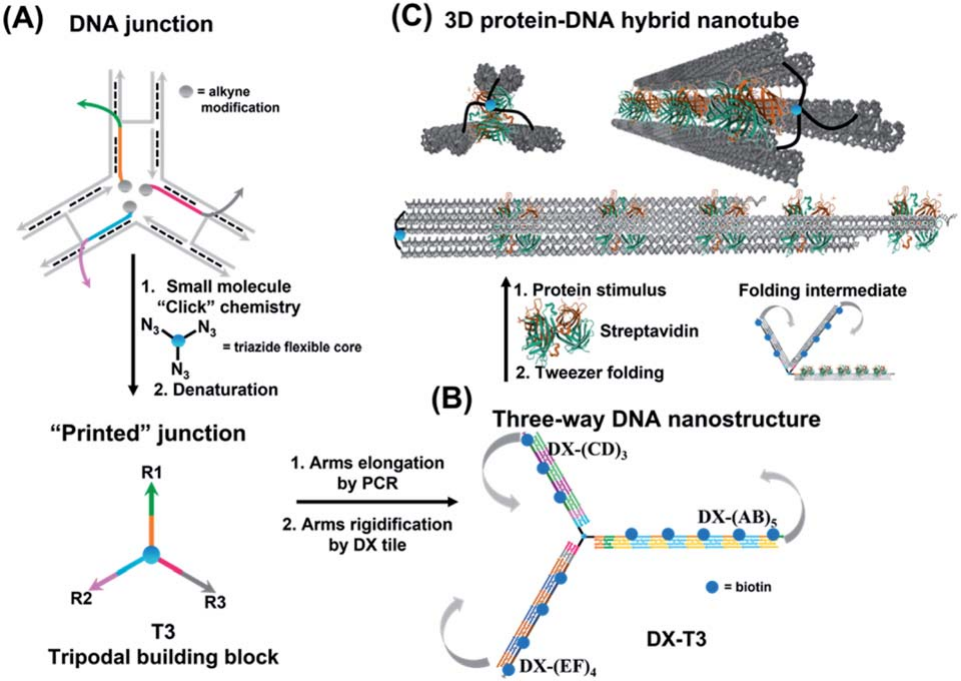
Schematic representation of three-way DNA nanostructure synthesis *via* (A) a “printing” strategy from a DNA junction, (B) arms elongation and rigidification, and (C) protein-binding tweezer formation. Biotinylated sequences were placed at an equidistant location from the branching core to favour binding to the same streptavidin. Upon incubation with streptavidin, the three-way DNA nanostructure recognizes the multivalent protein, causing the 3 arms of DX-T3 to fold into a 3D nanotube-like structure.

Herein, we report the synthesis of an extended DNA tweezer with a trivalent synthetic molecule as the core and three rigid and long DNA arms of different sequences (Fig. 1). Site-specific, equidistant placement of biotin moieties on the three arms causes them to fold together when a streptavidin target is added, in a large-scale motion over hundreds of nanometers. The result is a multivalent, hybrid protein–DNA nanotube-like structure, where the nanotube “rungs” are streptavidin, and the arms are DNA. The three-way DNA scaffold is built using a “printing” process developed in our laboratory,^40,41^ in which a pattern of three DNA arms is covalently transferred from a DNA nanostructure onto a small molecule, which then acts as the tweezer fulcrum. The sequence uniqueness of the three arms allowed their simultaneous extension by PCR using three different long DNA strands as templates, and the protein-binding three-way DNA nanostructure is formed when the arms are rigidified and addressed with binding ligands. Because of the ability to change the nature of the small molecule spacer on demand, we show that a more flexible molecular spacer gives a significantly higher yield of the folded streptavidin–DNA nanotube than a small and rigid aromatic spacer. Our methodology can be used as a new tool for the construction of DNA-minimal, stimuli-responsive architectures.

## Results and discussion

### Design parameters and tweezer formation

To form large multivalent DNA tweezers using a size-defined three-way DNA nanostructure, there are four key design requirements. First, the long arms of the structure must be rigid, to translate molecular recognition into a large, directed motion about the junction, and to avoid intramolecular binding around the protein. Second, the binding sites on each arm must be equidistant from the branched building block so that multiple arms of the structure bind to the same guest molecules. As a third criterion, we were interested in generating this structure from a small number of strands, which requires the use of identical DNA sequences in strategic positions. Fourth, the tweezer junction must be covalently connected to DNA arms of different sequences, so that the tweezer arms can be elongated using the polymerase chain reaction (PCR) which requires cycles of heating and cooling, and its structure and flexibility must be tunable.

Our group has recently developed methods for DNA “printing” – transferring a pre-defined pattern of DNA sequences onto other types of materials.^40^ This simple process was used to generate synthetic vertices attached to multiple DNA arms with controllable valency, different sequences, and directionalities.^40^ We applied this printing process to generate a small molecule aromatic core connected to 3 different DNA arms (Trimer or T3; DNA arms R1, R2, R3: 42, 39, and 41 bases respectively) (Fig. S1, ESI-VI).†

We were interested first in producing the long tripodal tweezer. We designed each of the three short arms of T3 to act as a forward primer for elongation by PCR. Three long DNA strands were required as PCR templates. It is possible to use the viral single-stranded DNA scaffolds that are normally employed for DNA origami,^42,43^ but this would then require a large number of complementary strands to rigidify the structure and substitute it with protein-binding units. To reduce the number of DNA components, we needed custom-made, long sequences with repeating DNA regions, as well as unique regions, and with full control over their number and placement. These sequences are too long to be built on an automated DNA synthesizer. We generated these template strands using a “temporal growth” method previously developed by our group.^44^ In this method, the strands are built by sequentially adding complementary DNA building blocks. Each building block is composed of a strand with the desired sequence, and a complementary strand is used to form sticky end overhangs at each end of the duplex. DNA building blocks are progressively added to a seed unit that can only hybridize in one direction with simultaneous enzymatic ligation to covalently attach each additional building block as it is introduced. PCR is then used to isolate and enrich the full-length product. Each building block can be designed with different sequence domains, giving different DNA patterns. This resulted in three strands with alternating repeating domains: (AB)5, (CD)3 and (EF)_4_, where A–F are 42 base–pair building blocks of different sequences (ESI-VII).† Another important advantage of this strategy over DNA origami is that structural dimensions can be larger, as they can take advantage of the full length of the template strands, rather than relying on folding of a scaffold strand.

**Fig. 2:**
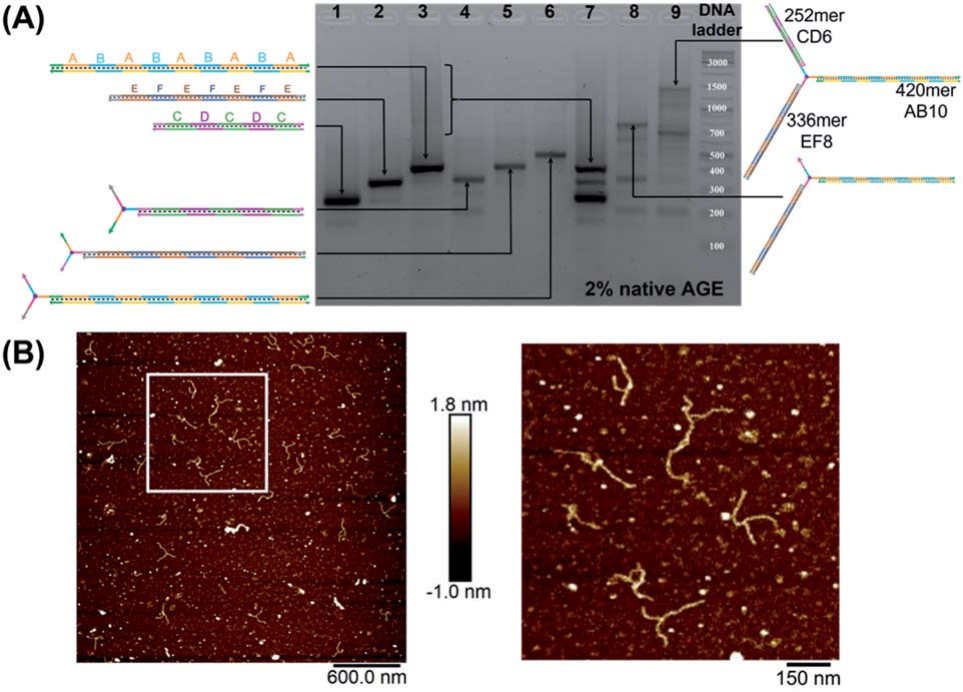
Native agarose gel electrophoresis (AGE) characterization of mono-, bi- and tri-elongated “printed” junction (T3) (A). Lanes 1, 2, 3: (CD)_3_ (252 bps), (EF)_4_ (336 bps) and (AB)_5_ (420 bps) respectively; lanes 4, 5 and 6: T3 separately elongated by PCR with (CD)_3_ (315 bps), (EF)_4_ (399 bps) and (AB)_5_ (483 bps), respectively. A–F are 42 base–pair building blocks of different sequences. Lanes 7 and 9: simultaneous PCR performed in the presence of (CD)_3_, (EF)_4_ and (AB)_5_ without T3 and with T3 (1071 bps), respectively. Lane 8: PCR product of (EF)_4_ and (AB)_5_ with T3 (819 bps). GeneRuler DNA ladder mix is used. (B) AFM characterization of “printed” junction with elongated arms in its double-stranded form. Note that the spherical structures are salts resulting from the surface drying, while the branched structures correspond to the extended junction.

We first elongated each of the three arms of T3 separately, using PCR with one of the three long strands as template, resulting in a clean product (Fig. 2A, native agarose gel electrophoresis (AGE) lanes 4, 5 and 6, ESI-VII†). Simultaneous extension of all three unique arms resulted in the formation of the desired product possessing three different arms with their expected lengths, in addition to side-products from elongation of one or two arms (Fig. 2A, native AGE lane 9). Band excision from this gel led to the tripodal branched DNA structure with elongated arms. Characterization by AFM showed formation of the correct product as a monodisperse, star-like structure with three long arms and varying angles between them. In this structure, the double-stranded arms have a high degree of flexibility (Fig. 2B) which may favor the intramolecular folding of the arms on themselves, impinging the tweezer effectiveness.

**Fig. 3:**
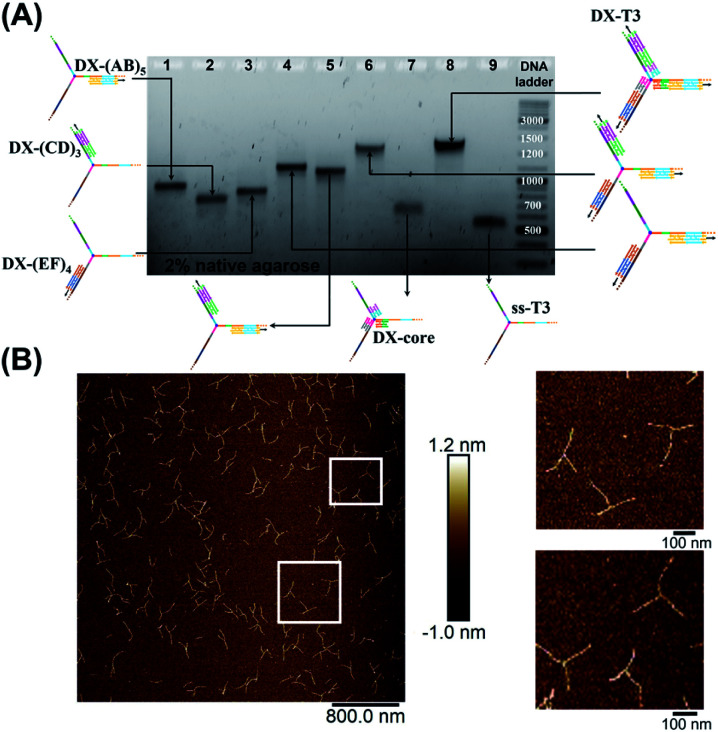
Stepwise assembly of three-way DNA nanostructure, using elongated single-stranded “printed” junction (ssT3) as a scaffold, checked by native AGE (A). Lanes 1, 2 and 3 correspond to DX tile staples assembled on (AB)_5_, (CD)_3_ and (EF)4, respectively. Lanes 4, 5, 6, 7 and 8 correspond to the assembly of DX tiles staples on (AB)_5_/(EF)_4_, (AB)_5_/(CD)_3_, (AB)_5_/(EF)_4_/(CD)_3_, core and (AB)_5_/(EF)_4_/(CD)_3_/core (DX-T3), respectively. Lane 9 corresponds to ssT3. GeneRuler DNA ladder mix is used. (B) AFM characterization of three-way DNA nanostructure in its DX-tile form (DX-T3). The population of fully assembled three-way DNA nanostructures calculated from AFM images is around 75% (*N* = 190). This percentage includes the interconnected structures resulting from sample deposition on the mica surface in addition to structures with one- and two-arms. Contour lengths of DX-tiles assembled on ssT3 corresponded well to the expected values (141 ± 26 nm for DX-(AB)_5_, 104 ± 27 nm for DX-(EF)_4_, and 86 ± 15 nm for DX-(CD)_3_) (ESI-IX).†

To rigidify our construct for subsequent tweezer formation and materials patterning, periodic double-crossover (DX) tile structures were designed to assemble on each of the arms (ESI-III. B2).† These tiles have an even number of helical half-turns between crossover points (DAE) and they consist of 1 helical turn (10 bps) between the crossover points and 1.5 helical turns (16 bps) on the outer arms of each domain. They also feature a specific 4 bp sticky-end interaction between the tiles, as this interaction provides further robustness and rigidity to the construct.^45^ First, we verified the clean formation of individual DX tile assemblies on the three template strands by annealing the component strands and templates from 95 to 4 °C over 4 h (non-denaturing AGE, Fig. S12, ESI-IX).† AFM images confirmed the formation of 1D structures with increased rigidity in comparison to double-stranded backbones and contour lengths that corresponded well to the expected values (130 ± 30 nm for DX-(AB)_5_, 102 ± 28 nm for DX-(EF)_4_, and 85 ± 24 nm for DX-(CD)_3_) (ESI-IX).† We then assembled these DX structures onto the tripodal branched structure in its single-stranded form ssT3 (Fig. S11, ESI-VIII†) by annealing it with all DX staple strands. Stepwise assembly of DX tiles on each arm (Fig. 3A) showed the formation of clean products in each case, and simultaneous assembly of DX tiles on the three arms of ssT3 was nearly quantitative, yielding a monodisperse product DX-T3. AFM images (Fig. 3B) revealed a higher rigidity of each individual arm in comparison to the double stranded version (Fig. 2B). The enhanced rigidity allows precise patterning of nanomaterials, such as nanoparticles or proteins with defined and constant separation, and the different sequences in each arm allow independent and selective hetero-patterning. Enhanced arm rigidity also favors the controlled folding of this large tweezer in response to an external stimulus.

### Selective streptavidin patterning

**Fig. 4:**
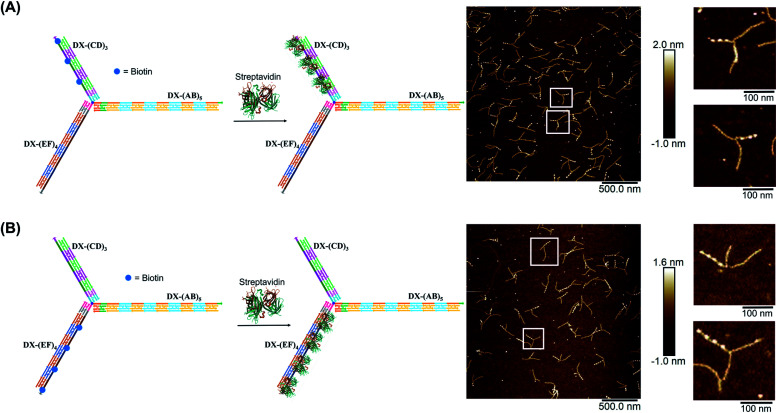
AFM characterization of three-way DNA nanostructure patterned with periodic streptavidin proteins. Patterning of (A) DX-(CD)_3_ arm and (B) DX-(EF)_4_ arm. The bright dots in the AFM images correspond to the patterned streptavidin. The population of structures with exactly three (A) and four (B) streptavidin calculated from AFM images is 76% (*N* = 186) and 77% (*N* = 120), respectively. This percentage excludes the interconnected structures resulting from sample deposition on the mica surface.

We demonstrated selective material organization on each arm of the elongated structure using biotin–streptavidin interactions.^46,47^ Each individual arm consists of two alternating, different building blocks (*e.g.*, A and B in (AB)_5_), and the repeating blocks have different sequences on the three arms. This allows the selective functionalization of biotin molecules at the building block of interest. Building block C was first functionalized with biotin, resulting in 3 periodic biotin units on the DX-(CD)_3_ arm. Upon addition of streptavidin, structures with exactly three proteins on the DX-(CD)_3_ arm were observed by AFM imaging, with more than 75% yield (Fig. 4A). Similarly, AFM images revealed that we were able to selectively pattern four streptavidin on DX-(EF)_4_ with a 77% yield (Fig. 4B). This approach can be further expanded to aperiodic and periodic patterning of proteins or nanomaterials within any elongated structure (*e.g*. 3-arm, 4-arm or 5-arm molecules) and with any user-defined sequence and length.

### Streptavidin induced tweezer folding

Finally, we explored our three-way DNA nanostructure as a nanotweezer capable of recognizing a multivalent protein. Streptavidin – as a tetrameric protein model – is capable of binding four different biotin molecules. We hypothesized that the addition of this multivalent protein would fold the 3 arms of DX-T3 into a 3D nanotube-like structure. DX tiles with biotinylated A, C, and E domains were thus assembled onto our tweezer scaffold and the entire construct was incubated with streptavidin. Biotinylated moieties were equidistant from the branching core to favor binding to the same streptavidin.

AFM images revealed the formation of populations with no streptavidin bound, streptavidin bound on multiple arms or crosslinking different structures, and the product of interest which is the linear/tubular structure (ESI-XI. Fig. S16†). The yield of the latter population was low (around 10%).

**Fig. 5:**
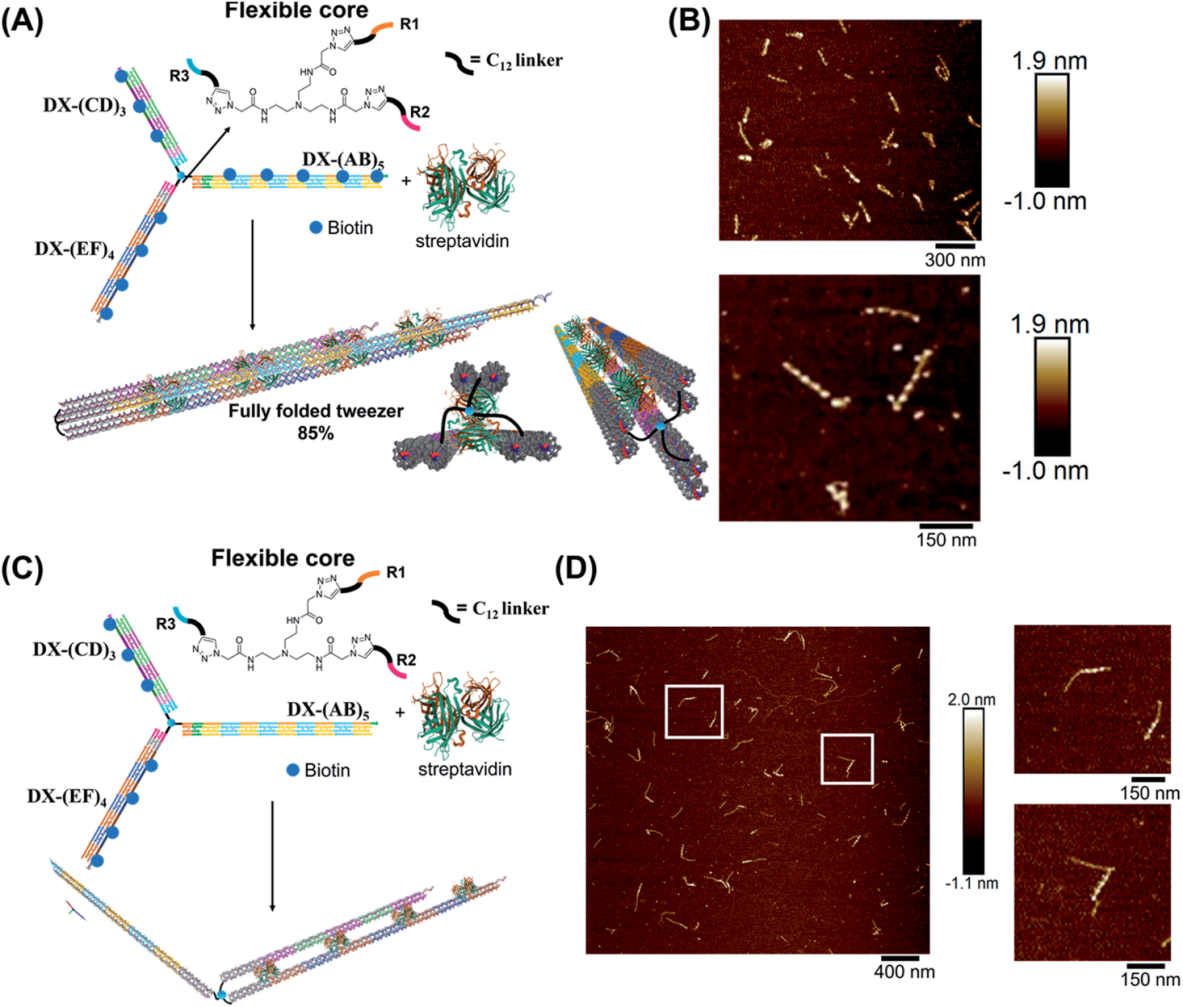
Schematic representation and AFM images of the tweezer folding. Folding of all arms, featuring periodic biotin moieties, in the presence of streptavidin into a tubular-like structure (schematic representation (A) and AFM images (B) (check ESI-XI,† for more AFM images)). Folding of DX-(CD)_3_ and DX-(EF)_4_ arms, featuring periodic biotin moieties on DX-(CD)_3_ and DX-(EF)_4_, in the presence of streptavidin (schematic representation (C) and AFM images (D)). The population of fully folded structures calculated from AFM images is 85% (*N* = 120) while that of the partially folded structure (DX-(CD)_3_ and DX-(EF)_4_ arms is 75% (*N* = 90). This percentage excludes the interconnected structures resulting from sample deposition on the mica surface.

We hypothesized that the junction spacer of our structure might be too rigid and small to allow the efficient folding of the tweezer onto the protein units. We thus generated another asymmetric DNA-small molecule trimer where the tri-functionalized phenyl core was substituted with a more flexible tertiary amine core, and connected to the DNA arms *via* hexaethylene linkers to provide further flexibility (f-T3 for flexible T3, ESI-IV.B and ESI-XII†).^48^ This more flexible core played a major role in improving the bending degree of the arms in the DNA tweezer. As shown in Fig. 5A and B, the population of the tubular structures drastically increased from 10%, reported for the rigid core, to 85% (*N* = 120) with the flexible core. In the current design, the first biotin moiety is located at an approximate distance of 27 nm (84 bps), while the flexible spacer only adds around 2 nm. The flexibility of the linker is thus most likely a major contributor to the enhancement in the tweezer folding, although we do not exclude the possibility of the additional spacer playing a role. We speculate that the lower limit for the distance between the biotin moiety and the branching core unit is 5 nm (the approximate size of streptavidin), but sterics may result in a higher value. To measure the core flexibility and estimate the degree of motion of the DNA arms as they close around the target proteins, we built an automated counting software that analyzes the angles between the arms from the AFM images of all the constructs (ESI-X†). Since the initial, rigid small molecule core consists of 1,3,5-tris(azidomethyl)benzene, the ideal angle between each of the arms would be 120°, and deviation from this angle may be used as an indirect measure of core flexibility. This assumption is complicated by deposition and drying on the AFM substrate, but we reasoned that the histograms of the angles in the structures, compared to each other, would inform on the rigidity/flexibility of the core (ESI-XII†). The flexible core resulted in a slightly larger standard deviation than the rigid core. Even though the standard deviation difference is low, the core's flexibility had a drastic effect on the efficiency of tweezer folding. Assuming a junction angle of ∼120° between the arms, the average distance between the ends of DX-AB and DX-EF arms is ∼213 nm, therefore the tweezer arms would have travelled a large distance of around 106 nm between the open and closed forms (ESI-XII).†

To further investigate the folding of our DNA tweezer, assembled using the flexible core, we functionalized only building blocks C and E (and not A) with biotin, and incubated the construct with streptavidin. AFM revealed the periodic pattering of four streptavidin units, with merging of two arms and an unbound DX-AB arm (Fig. 5C and D). This indicates that our strategy can be applied to specifically fold any arm of the three-way DNA nanostructure depending on the position of biotin units and the needed application. On the other hand, and as detected by AFM, we have a small population of the partially folded structure, DX-(CD)3 and DX-(EF)4 arms with 2 streptavidin units, indicating that 2 streptavidin units may be enough to lock the tweezer in its folded form. The flexibility of the core, coupled with the rigidity of the arms are major determinants of proper tweezer folding, as they will bring biotin units on different arms in close enough proximity for streptavidin to bind to them. Once a single streptavidin is bound, the other biotin moieties will be close to each other and thus it may be easier for the next streptavidin to simultaneously bind to the other arms as the entropic cost is reduced. Despite the pre-organization afforded by the first binding event, folding the tweezer is entropically costly, and the strength of biotin–streptavidin binding enthalpically offsets this cost. Future protein–ligand pairs will require binding that overcomes this entropic cost. This can be controlled by increasing the number of binding sites to the protein.

Examination of the height profile of the DNA segment located between two consecutive streptavidin molecules revealed an average height of 1.1 ± 0.2 nm for the structures having one streptavidin-templated arm (Fig. 4). Similar DNA heights (1.2 ± 0.2 nm) were observed for branched structures having two arms DX-CD and DX-EF held together by streptavidin (Fig. 5D). In contrast, an average DNA height of 2.2 ± 0.4 nm was observed between two consecutive streptavidin units in the structures that have all three arms folded together (Fig. 5B). This 80% height increase in the latter case supports the folding of the three tweezer arms into a 3D-protein/DNA nanotube, where the streptavidin moieties are surrounded by DNA tiles. Our DNA arms thus fold around their protein targets, providing stimuli-responsive behavior that propagates large-scale motion and assembly reconfiguration. We envision that the appropriation of our technology to diverse and multiple multivalent proteins will proffer applications in sensing and biological diagnostics, wherein several identical or different proteins are recognized simultaneously to generate sensitive read-outs.

## Conclusions

We have developed a versatile method for the assembly of a large DNA nanotweezer with multiple, asymmetric arms. Using DNA strands with chemically conjugated branched units, we imbue our construct with flexibility and asymmetry that is propagated by the sequential growth of all three unique DNA arms. The dynamic behavior of our core junction, coupled with the rigidity of DNA arms, is critical to ensure the folding of our tripodal nanotweezer in response to protein stimuli, yielding a new DNA nanostructure: a 3D protein–DNA hybrid nanotube. Arm rigidity also allows precise nanomaterial organisation with defined separation, and the unique sequences in each arm allow independent and selective asymmetric streptavidin patterning. This method is complementary to DNA origami, but it is advantageous when larger wireframe structures are desired, whose size is not limited by folding of a viral scaffold strand; because it requires significantly fewer component strands, it is also valuable when a structure needs to be built from a minimal number of starting strands, such as *in vivo* applications. The covalent nature of our tweezer fulcrum endows robustness of the structure for biological applications, facilitates PCR manipulations and is highly tunable because of the variety of small molecules that could be used as corners of different strands. Our methodology will find broad applicability in generating more complex DNA-hybrid materials, especially in conjunction with other types of multivalent proteins or nanoparticles. By applying our new “printing-elongation-folding” methodology to more diverse small molecule cores with further branching degrees and trigger stimuli, we will export the utility of hybrid DNA-small molecule motifs to the construction of extended DNA nanotweezers with more diverse and complex guest sensing behaviors.

## Data availability

All experimental data, and detailed experimental procedure are available in the ESI.†

## Author contributions

D. S. helped design the project, primarily contributed to the production of experimental data, and wrote the manuscript. T. T. helped in the synthesis of the branched DNA molecule and acquired most of the AFM images. C. L.-B. carried out the automated angle counting. A. L. P. synthesized the flexible azido functionalized branching core. F. J. R. helped edit the manuscript. D. R. synthesized alkyne modification for DNA synthesis. H. F. S. designed the project, guided the interpretation of data and result discussion, co-wrote the paper, and provided funding for the project.

## Conflicts of interest

There are no conflicts to declare.

## Supplementary Material

SC-013-D1SC04793K-s001
