# Supplementary material for: Asymmetric patterning drives the folding of a tripodal DNA nanotweezer
Source: Chem Sci. 2021 Nov 16;13(1):74–80. doi: 10.1039/d1sc04793k (PMC8694393; doi:10.1039/d1sc04793k)
Supplement: SC-013-D1SC04793K-s001 [file SC-013-D1SC04793K-s001.pdf]

Electronic Supplementary Material (ESI) for Chemical Science.
This journal is © The Royal Society of Chemistry 2021

Supporting Information

Asymmetric patterning drives the folding of a tripodal DNA nanotweezer

Daniel Saliba[+], Tuan Trinh[+], Christophe Lachance-Brais, Alexander L. Prinzen, Felix J. Rizzuto, Donatien de Rochambeau, and Hanadi Sleiman*

Department of Chemistry, McGill University, 801 Sherbrooke Street West, Montreal, QC H3A 2K6, Canada.

Table of Contents

SI-I. Materials 2
SI-II. Instrumentation 2
SI-III. Oligonucleotide strands: synthesis, purification, and sequences 3
A. DNA synthesis and purification 3
1. Non-modified DNA sequences 3
2. Alkyne- and biotin- modified DNA sequences 3
B. DNA sequences 4
1. Sequences for DNA junction, temporal growth, and PCR experiments 4
2. Sequences for periodic DX-tile assemblies 5
SI-IV. Synthesis of triazide molecule core and alkyne-modified phosphoramidite 7
A. Synthesis of alkyne-modified amidite: 7
B. Synthesis of triazide molecule core: 7
SI-V. Mass spectrometry characterization of reactive DNA sequences 8
SI-VI. Assembly of DNA junction, "printed" junction (T3) construction and isolation 9
SI-VII. Temporal growth and "printed" junction (T3) incorporation by PCR 10
SI-VIII. Conversion of ds backbones and three-way DNA scaffold into ssDNA 13
SI-IX. DX tile assemblies 14
SI-X. Automated angle counting 16
SI-XI. Nanotweezer formation and folding in response to streptavidin 17
SI-XII. Angle distribution of three-way DNA nanostructures with rigid and flexible core 19
SI-XIII. AFM microscopy 20
References 20

SI-I. Materials

Acetic acid, magnesium chloride hexahydrate, sodium chloride, sodium citrate, sodium hydroxide, dimethyl sulfoxide (DMSO), copper (II) sulfate ($CuSO_4$) and tris(3hydroxypropyltriazolylmethyl) amine (THPTA), tris(2-aminoethyl)amine, 2-azidoacetic acid, N,N'-dicycolhexylcarbodiimide were used as purchased from SigmaAldrich. 1 µmol Universal 1000Å LCAA-CPG solid supports and reagents for automated DNA synthesis were used as purchased from BioAutomation. Acrylamide (40%)/bisacrylamide 19:1 solution, tris(hydroxymethyl)-aminomethane (Tris), urea, EDTA, ammonium persulfate and tetramethylenediamine and agarose were obtained from BioShop. GelRedTM (Biotium, Inc.) was purchased from VWR. Glacial acetic acid, adenosine triphosphate (ATP), hydrochloric acid, SYBR® safe and SYBR gold DNA gel stains were purchased from Thermofisher. Sephedex desalting columns (Gel-Pak™ 2.5) were purchased from Glen Research. AFM cantilevers (SCANASYST-AIR) were purchased from Bruker. RubyRed mica was purchased from Electron Microscopy Sciences. Kits for T4 PNK and Quick T4 DNA ligase were purchased from New England Biolabs. MyTaq HS Red mix (Bioline) was purchased from FroggaBio. QIAquick gel extraction, PCR purification and nucleotide purification kits from Qiagen were used for extraction or cleanup of PCR and "printed" junction products. O'GeneRuler Ultra Low Range DNA ladder and GeneRuler DNA ladder mix were purchased from Thermofisher.

1xTAMg buffer is composed of 45 mM Tris and 12.5 mM $MgCl_2.6H_2O$ with pH adjusted to 8.0 using glacial acetic acid. 1xTBE buffer is composed of 90 mM Tris, 90 mM boric acid and 2 mM EDTA with a pH of 8.3. 1xSSC buffer is composed of 150 mM sodium chloride and 15 mM EDTA with a pH adjusted to 7.0 with a few drops of 1M HCl. 1xTAE buffer is composed of 40 mM Tris, 20 mM Acetate and 1 mM EDTA with a pH around 8.6. 1xALK buffer is composed of 30 mM sodium hydroxide and 2 mM EDTA. TEAA mobile phase is 50 mM triethylammonium acetate with pH adjusted to 8.0 using glacial acetic acid.

SI-II. Instrumentation

The oligonucleotide sequences were synthesized via standard automated oligonucleotide solid-phase synthesis on a BioAutomation MerMade MM6 DNA synthesizer at 1 µM scale. Polyacrylamide gel electrophoresis (PAGE) was employed (20 x 20 cm vertical Hoefer 600 electrophoresis unit) to purify crude products with a length less than 100 bps, while agarose gel electrophoresis (AGE) was carried out on an Owl Mini gel electrophoresis unit for purifying products with a length more than 100 bps. Gel images were aquired using a ChemiDocTM MP System from Bio-Rad Laboratories. UV-Vis quantifications (OD260) were performed with a NanoDrop Lite Spectrophotometer and using IDT's

molar extinction coefficient at 260. Thermal annealing of DNA nanostructures, enzymatic reactions, and polymerase chain reaction (PCR) were conducted using an Eppendorf Mastercycler® 96 well thermocycler. Liquid Chromatography Electrospray Ionization Mass Spectrometry (LC-ESI-MS) was performed using Dionex Ultimate 3000 coupled to a Bruker MaXis Impact™ QTOF. Electroelution was carried out on an Elutrap Electroelution System from Whatman®. Atomic force microscopy (AFM) images were acquired using a Multimode 3 scanning probe microscope and Nanoscope V controller (Bruker, Santa Barbara, CA).

SI-III. Oligonucleotide strands: synthesis, purification, and sequences

A. DNA synthesis and purification

1. Non-modified DNA sequences

All clip strands were synthesized using Mermade MM6 DNA synthesizer at 1 µmol scale. The coupling efficiency was checked by the removal of 5’ DMT groups. The strands were deprotected and cleaved from the solid support in the presence of concentrated ammonium hydroxide solution after which PAGE (8-20% polyacrylamide/8M urea) was employed for purification. After electrophoresis, the desired band was excised, crushed, and incubated overnight in 10 mL of autoclaved water at 65 °C. The sample was then concentrated down to ~1 mL, desalted using size exclusion chromatography (Sephadex desalting columns), and quantified by Nanodrop.

2. Alkyne- and biotin- modified DNA sequences

For alkyne-functionalized DNA: DMT protected alkyne amidite (Section SI-IVA) was dissolved in anhydrous acetonitrile, activated with equivolume of 0.25 M 5-(ethylthio)tetrazole and mixed with the solid support for 10 minutes. This extended coupling time, performed in a glove box under argon atmosphere, was applied to ensure a high coupling efficiency. 3% dichloroacetic acid in dichloromethane was used to remove the DMT protecting group of the alkyne’s amidite. Next, the solid support was treated with concentrated aqueous ammonium hydroxide solution for 12 hours at 65 ^{o}C. The solution was evaporated, under reduced pressure at 60 ^{o}C, dissolved with autoclaved water and filtered by 0.22 µm centrifugal filter before purifying by Reversed Phase High Performance Liquid Chromatography (RP-HPLC), (3-30% acetonitrile in 50 min).

For biotinylated DNA sequences: 5’ dT biotin phosphoramidite coupling was performed on the DNA synthesizer using standard protocol. The solid support was treated with concentrated aqueous ammonium hydroxide solution for 12 hours at 65 ^{o}C followed by purification using RP-HPLC.

B. DNA sequences

1. Sequences for DNA junction, temporal growth, and PCR experiments

The schematic representation of assembly of a temporal growth backbone is summarized as follows:

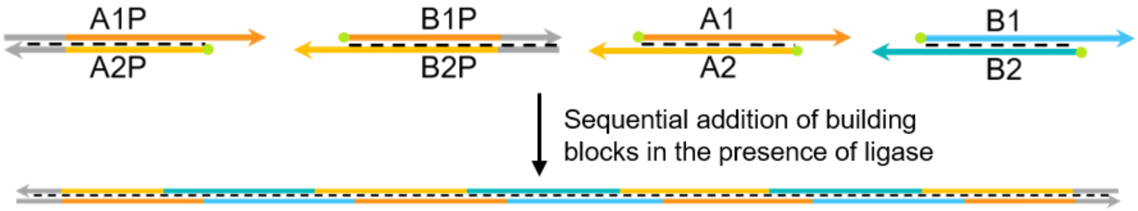

Figure S1. Nomenclature of DNA sequences used to form the temporal growth backbone with A and B repeating domains. Similar nomenclature is used for the backbones with C, D and E, F domains. A2P, B1P, A1, A2, B1 and B2 sequences are phosphorylated (green dot at 5' end). Sequential addition of building blocks, in the presence of quick ligase, results in generating a user-defined sequence and length backbone with repeating domains.

Table S1. Oligonucleotide sequences of different temporal growth backbones and DNA junction. All sequences are listed from 5' to 3' (Alk = alkyne-modified phosphoramidite).

Name	Sequence (5' → 3')
**A1P**	AGGTTAGTGGCGATCAGAAGAAATCTGGCTGCGCTTGAAACAACGGAAGGTCAT GCTTTAGGA
**A2P**	TGACCTTCCGTTGTTTCAAGCGCAGCCAGATTTCTTCTGATCGCCACTAACCT
**B1P**	AATCTGGCTGCGCTTGAAACAACGGAAGGTCATGCTTTAGGAGAATAGCACATC TAGACTGGT
**B2P**	ACCAGTCTAGATGTGCTATTCTCCTAAAGCATGACCTTCCGTTGTTTCAAGCGCA GCCAGATTTCTTCTGATC
**A1**	AATCTGGCTGCGCTTGAAACAACGGAAGGTCATGCTTTAGGA
**A2**	TGACCTTCCGTTGTTTCAAGCGCAGCCAGATTTCTTCTGATC
**B1**	ATCAAACCAAAGTTCAGCAACAGGCCGTTAAGGATCAGAAGA
**B2**	CTTAACGGCCTGTTGCTGAACTTTGGTTTGATTCCTAAAGCA
**C1P**	CACAGCCGCGAAGATCCGCTTTACCACATTCGAGGCACGATGTACGTCCACACTT GGAACCTC
**C2P**	GTGTGGACGTACATCGTGCCTCGAATGTGGTAAAGCGGATCTTCGCGGCTGTG
**D1P**	TACCACATTCGAGGCACGATGTACGTCCACACTTGGAACCTCCTATGACGATGGCGTTC AATA
**D2P**	TATTGAACGCCATCGTCATAGGAGGTTCCAAGTGTGGACGTACATCGTGCCTCGAATGT GGTATGACATAGCA
**C1**	TACCACATTCGAGGCACGATGTACGTCCACACTTGGAACCTC
**C2**	GTGTGGACGTACATCGTGCCTCGAATGTGGTATGACATAGCA
**D1**	ATCGCACATCCGCCTGCCACGCTCTTAACGTATGCTATGTCA
**D2**	TACGTTAAGAGCGTGGCAGGCGGATGTGCGATGAGGTTCCAA
**E1P**	CTGGAGTCAACGCATCGATCTTCATAAATTGTGAGCTACCTGGCTCATGCCAACGTCCG CAAC
**E2P**	TTGGCATGAGCCAGGTAGCTCACAATTTATGAAGATCGATGCGTTGACTCCAG
**F1P**	TCATAAATTGTGAGCTACCTGGCTCATGCCAACGTCCGCAACTTCGTCCGAATTCACCTG CAA
**F2P**	TTGCAGGTGAATTCGGACGAAGTTGCGGACGTTGGCATGAGCCAGGTAGCTCACAATTT ATGACAGTCCGTGG
**E1**	TCATAAATTGTGAGCTACCTGGCTCATGCCAACGTCCGCAAC
**E2**	TTGGCATGAGCCAGGTAGCTCACAATTTATGACAGTCCGTGG
**F1**	TGGACTGATATCACTACACAGAAGACAATCCTCCACGGACTG

**F2**	AGGATTGTCTTCTGTGTAGTGATATCAGTCCAGTTGCGGACG
**Primer AB (forward)**	AGGTTAGTGGCGATCAGA
**Primer AB (reverse)**	ACCAGTCTAGATGTGCTATTCT
**Primer CD (forward)**	CACAGCCGCGAAGAT
**Primer CD (reverse)**	TATTGAACGCCATCGTCATAG
**Primer EF (forward)**	CTGGAGTCAACGCATCG
**Primer EF (reverse)**	TTGCAGGTGAATTCGGAC
**W1AB**	AAATCTCGAACACATTTATATGGTCAACTGAAAAAAAAAAGTAATACCAGATGGTTCTA GTCGGCACTTC
**W2HE**	TTAACCGGCGGCCTTTTCTTCTATACTGGCAAAAAAAAAACAGGATTAGCAGAGTTACA ACCAATGGCTT
**W3DI**	AGATAGTGTACCGCTTTGGCCTTGGTCCATAAAAAAAAAAACCGCGACTGCGAGTTTGC GCCACACCGTA
**D1**	GAAGTGCCGACTAGAGGCCGCCGGTTAA
**D2**	AAGCCATTGGTTGTGCGGTACACTATCT
**D3**	TACGGTGTGGCGCATGTGTTCGAGATTT
**Alk-AT-primerAB (R1)**	Alk-GCCCGCTTTTCAGTTGACCATATAAGGTTAGTGGCGATCAGA
**Alk-DT-primerCD (R2)**	Alk-GTGCGGTTTTATGGACCAAGGCCACACAGCCGCGAAGAT
**Alk-HT-primerEF (R3)**	Alk-ACAGCGTTTTGCCAGTATAGAAGACTGGAGTCAACGCATCG
**TB**	CCATCTGGTATTACTTTTT
**TE**	TCTGCTAATCCTGTTTTT
**TI**	CGCAGTCGCGGTTTTTT

2. Sequences for periodic DX-tile assemblies

The oligonucleotide sequences used in the assembly of DX tiles along with their nomenclature and schematic representation are summarized as follows:

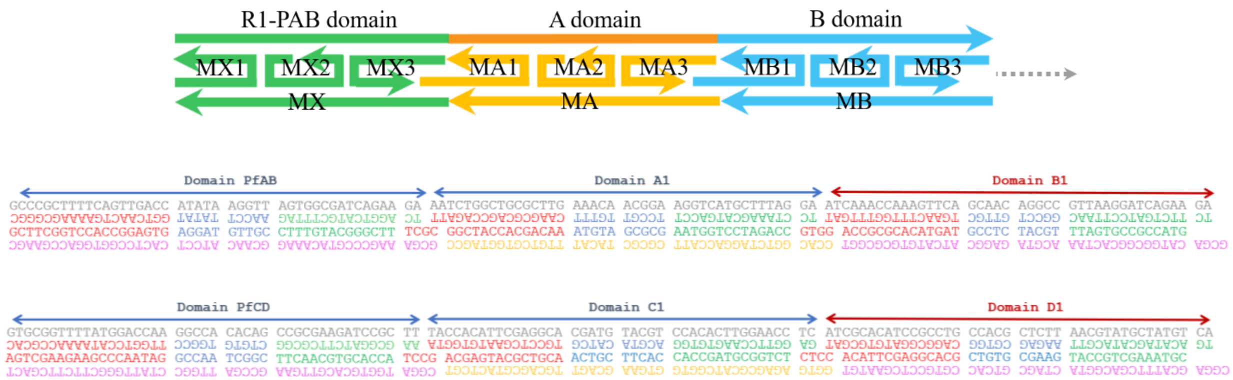

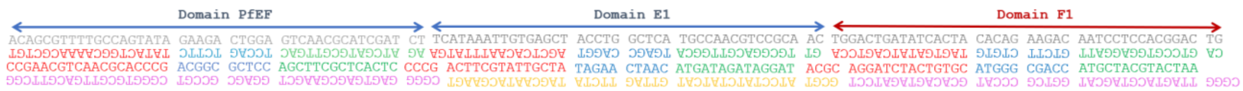

Figure S2. DAE-E DNA tiles used to form different DX tiles and their nomenclature. Sequences and structure of **DX-AB**, **DX-CD** and **DX-EF** tiles showing one of each of the 'AB', 'CD' and 'EF' repeating domains of the templating backbones, in addition to the DX tiles sequences of the unique DNA domain that is attached to the small molecule core forming **T3**. R1-PAB domain corresponds to the 42 bps domain that is attached to the small molecule core and including the primer region (primer AB (PAB)) that is responsible for PCR.

Table S2. Oligonucleotide sequences of different periodic DX tile staple strands. All sequences are listed from 5' to 3' (biot dT = biotin dT CED phosphoramidite).

**Name**	**Sequence (5' → 3')**
**MA**	CCACGGTCTAGGACCATTCGCGCTACATTTGTCGTGGTAGCC
**MA1**	TCGCGGCTACCACGACAACAAGCGCAGCCAGATT
**MA2**	TGTTTATGTAGCGCGTCCGT
**MA3**	Biot dT/TCCTAAAGCATGACCTAATGGTCCTAGACC
**MB**	GCGACATGGCGGCACTAAACGTAGAGGCATCATGTGCGCGGT
**MB1**	GTGGACCGCGCACATGATTGAACTTTGGTTTGAT
**MB2**	GTTGCGCCTCTACGTGGCCT
**MB3**	TCTTCTGATCCTTAACTTAGTGCCGCCATG
**R1-Pf-MX1**	GCTTCGGTCCACCGGAGTGGGTCAACTGAAAAGCGGGC
**R1-Pf-MX2**	TATATAGGATGTTGCAACCT
**R1-Pf-MX3**	TCAGGTCATGCTTTAGCTTTGTACGGGCTT
**R1-Pf-MX**	GCGAAAGCCCGTACAAAGGCAACATCCTCACTCCGGTGGACCGAAGC
**MC**	GGTGAGAGCGCATCGGTGGTGAAGCAGTTGCAGCGTACTCGT
**MC1**	TCCGACGAGTACGCTGCATGCCTCGAATGTGGTA
**MC2**	CATCGACTGCTTCACACGTA
**MC3**	Biot dT/GAGGTTCCAAGTGTGGCACCGATGCGCTCT
**D**	CGGAGCATTTCGACGGTACTAGCGTCACTGCTGGACTAAGCC
**MD1**	CTCCGGCTTAGTCCAGCACAGGCGGATGTGCGAT
**MD2**	CGTGGGTGACGCTAGAAGAG
**MD3**	TGACATAGCATACGTTTACCGTCGAAATGC
**R3-Pf-MX1**	AGTCGAAGAAGCCCAATAGTTGGTCCATAAAACCGCAC
**R3-Pf-MX2**	TGGCCGCCAATCGGCCTGTG
**R3-Pf-MX3**	AAGCGGATCTTCGCGGTTCAACGTGCACCA
**R3-Pf-MX**	CGGATGGTGCACGTTGAAGCCGATTGGCCTATTGGGCTTCTTCGACT
**E**	GCGTATCCTATCTATCATGTTAGTTCTATAGCAATACGAAGT
**ME1**	CCCGACTTCGTATTGCTAAGCTCACAATTTATGA
**ME2**	CAGGTTAGAACTAACTGAGC
**ME3**	Biot dT/GTTGCGGACGTTGGCAATGATAGATAGGAT
**F**	CGGGTTAGTACGTAGCATGGTCGCCCATGCACAGTAGATCCT
**MF1**	GCGCAGGATCTACTGTGCTAGTGATATCAGTCCA
**MF2**	CTGTGATGGGCGACCGTCTT
**MF3**	CAGTCCGTGGAGGATTATGCTACGTACTAA
**R5-Pf-MX1**	CCGAACGTCAACGCACCCGTATACTGGCAAAACGCTGT
**R5-Pf-MX2**	TCTTCACGGCGCTCCTCCAG
**R5-Pf-MX3**	AGATCGATGCGTTGACAGCTTCGCTCACTC
**R5-Pf-MX**	CGGGGAGTGAGCGAAGCTGGAGCGCCGTCGGGTGCGTTGACGTTCGG

SI-IV. Synthesis of triazide molecule core and alkyne-modified phosphoramidite

A. Synthesis of alkyne-modified amidite:

The DMT protected alkyne phosphoramidite (3-((3-(bis(4-methoxyphenyl)(phenyl)methoxy) propyl)(prop-2-yn-1-yl)amino)propyl(2cyanoethyl) diisopropylphosphoramidite) was synthesized by a literature procedure[1].

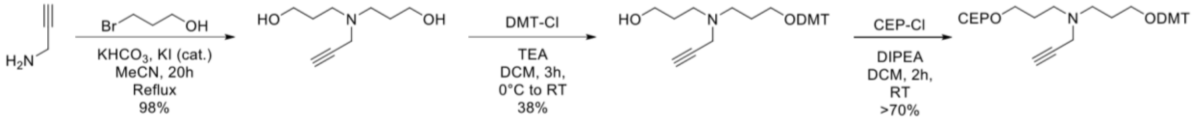

Figure S3. Synthesis of DMT protected alkyne-modified phosphoramidite.

B. Synthesis of triazide molecule core:

Tris(2-aminoethyl)amine (73 mg, 0.72 mmol) and 2-azidoacetic acid (200 mg, 2.87 mmol) were dissolved in DMF (20 mL). To this was added N,N'-dicyclohexylcarbodiimide (296 mg, 3.01 mmol) and the mixture was reacted for 24 h. The solution was then filtered to remove the insoluble urea and diluted with H_2O (100 mL) and extracted with ethyl acetate (3 × 100 mL). The organic phase was washed with saturated $NaHCO_3$ (200 mL) and saturated NaCl (200 mL) and then dried on $MgSO_4$, filtered and concentrated *in vacuo*. The crude material was purified by column chromatography (CH_2Cl_2/CH_3OH 9:1) to provide Compound **1** as a white powder (283 mg, 40%). ^{1}H NMR (500 MHz, $CDCl_3$): δ = 2.50 (s, 12H), 3.85 (s, 6H), 8.04 (s, 3H) ^{13}C NMR (125 MHz, $CDCl_3$): δ = 25.58, 37.22, 50.90, 53.45, 167.75, 172.94. HRMS (EI): calc. for $[C_{12}H_{22}O_3N_{13}]^+$ $[M+H]^+$: 396.19, found 396.1952.

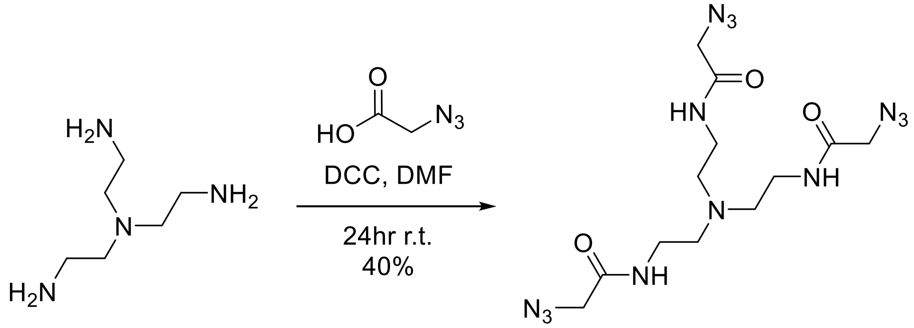

1

Figure S4. Synthesis of triazide flexible molecule core.

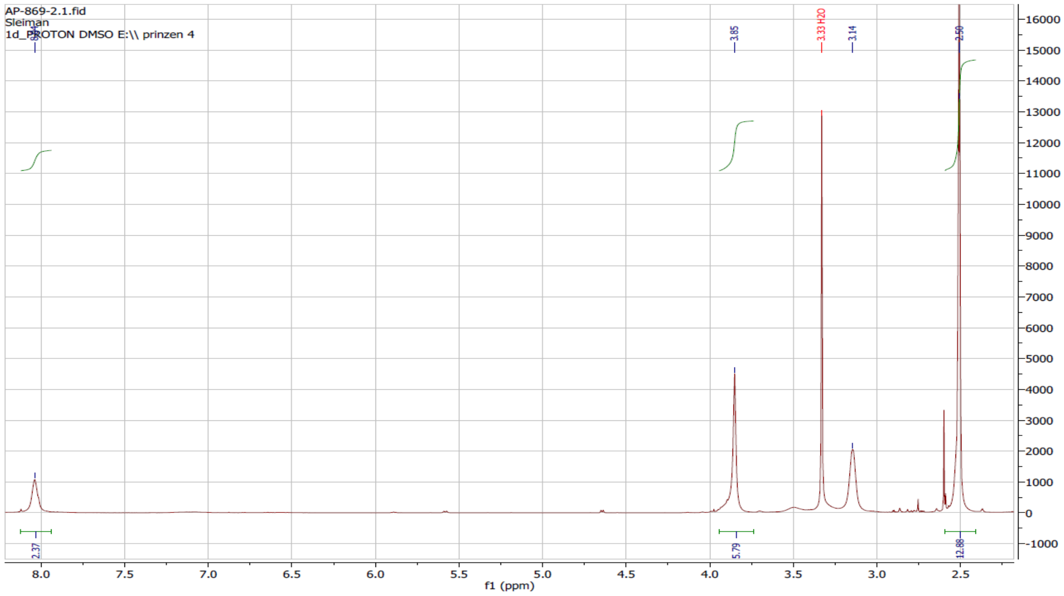

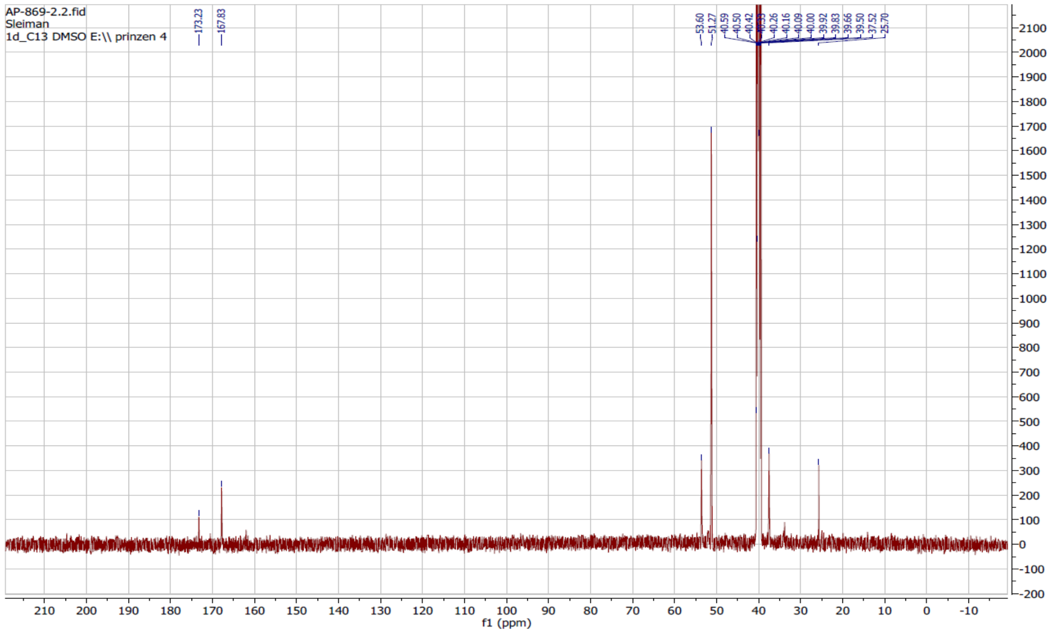

Figure S5. ^{1}H NMR (top figure) and ^{13}C NMR (bottom figure) spectra of triazide flexible core. Spectra were obtained in $CDCl_3$.

SI-V. Mass spectrometry characterization of reactive DNA sequences

The MS spectra of the alkyne reactive sequences, namely Alk-AT-primerAB, Alk-DT-primerCD and Alk-HT-primerEF with a single C12 spacer, revealed efficient couplings of the C12 spacer and alkyne amidites.

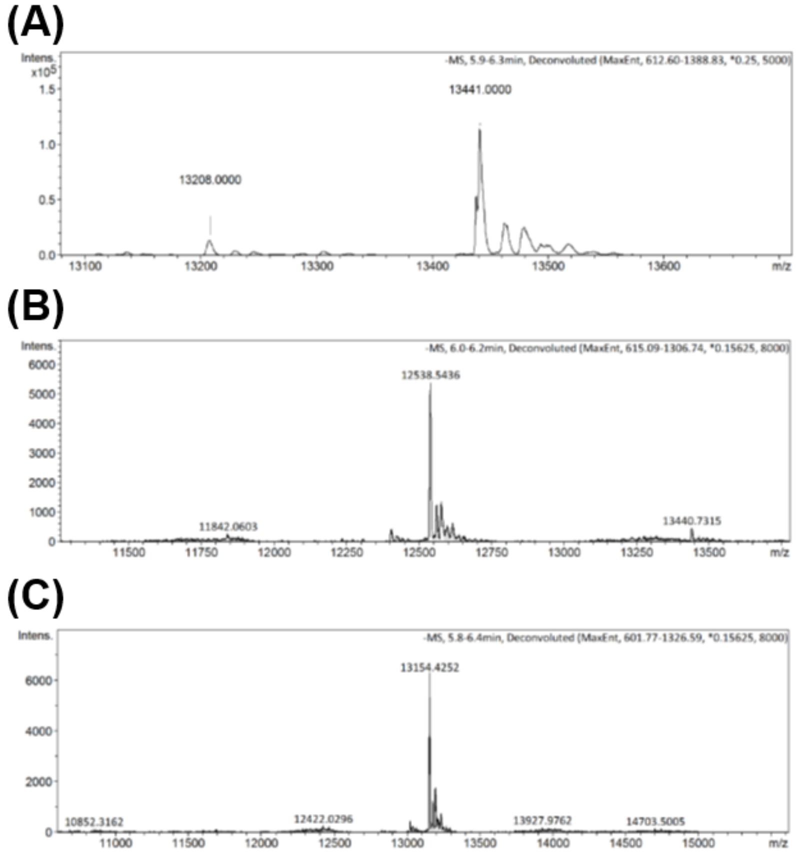

Figure S6. MS spectra of Alk-AT-primerAB (R1) where the calculated mass is 13416.3 and the mass found is 13441.0 (A), of Alk-DT-primerCD (R2) where the calculated mass is 12513.7 and the mass found is 12538.5 (B) and of Alk-HT-primerEF (R3) where the calculated mass is 13130.2 and the mass found is 13154.4.

SI-VI. Assembly of DNA junction, "printed" junction (T3) construction and isolation

The DNA junction was assembled using 12 DNA sequences, namely W1AB, W2HE, W3DI, D1, D2, D3, TB, TE, TI, in addition to the alkyne-functionalized sequences Alk-AT-primerAB (R1), Alk-DT-primerCD (R2), and Alk-HT-primerEF (R3) (Fig. S7).[1] The mixture was annealed from 95 ^{o}C to 4 ^{o}C for 4 hours, in 1xTAMg at a final concentration of 1 µM. The assembly was checked by 5% native PAGE which revealed one single major band, indicating the proper assembly of the DNA junction (Fig. S8 (A)). Next, the triazide molecule, dissolved in DMSO ($C_{initial}$ = 3 mM, C_{final} = 9 µM), was added to the DNA junction, along with 10 v/v% of $CuSO_4$/THPTA ($C_{initial(Cu)}$ = 1 mM, $C_{initial(THPTA)}$ = 5 mM) and 10 v/v% sodium ascorbate ($C_{initial}$ = 60 mM). The reaction mixture was incubated at room temperature for 2 hours and analyzed by denaturing PAGE. Denaturing PAGE revealed the formation of "printed" junction including 3 different primer regions (Fig. S8 (B)). The band corresponding to the "printed" junction was excised and the product was isolated by electroelution. The electroelution was performed in 0.5x TBE at 300 V for 3 hours after which the sample was purified using a nucleotide removal KIT from Qiagen.

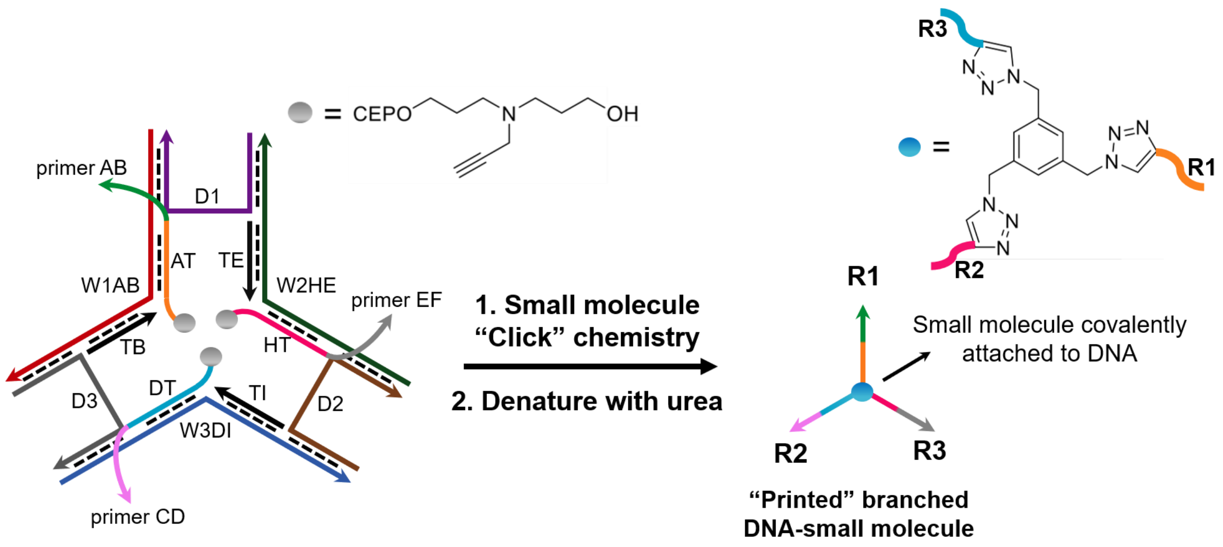

Figure S7. Schematic representation of the overall design approach of DNA junction and chemical structure of the alkyne reactive sequence. The sticky ends located at the 3' end of alkyne-reactive sequences represent the forward primer sequences. Sequences AT, DT and HT present at their 3' ends AB, CD and EF forward primer sequences.

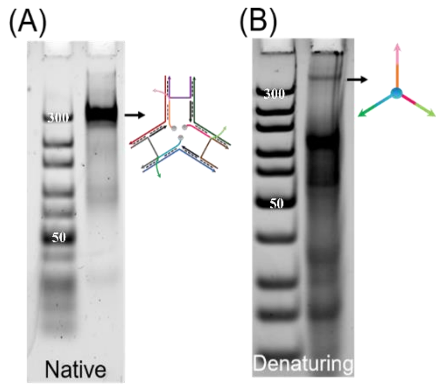

Figure S8. Native PAGE characterization of DNA junction assembly (A) and denaturing PAGE characterization of "printed" junction **(T3)** (B). O'GeneRuler Ultra Low Range DNA ladder is used in both gels.

SI-VII. Temporal growth and "printed" junction (T3) incorporation by PCR

Synthesis of temporal growth backbones with repeating domains was achieved following a previous reported protocol[2]. In summary, all component sequences with internal 5' ends (A2P, A1, A2, B1 and B2 for backbone AB, C2P, C1, C2, D1 and D2 for backbone CD and E2P, E1, E2, F1 and F2 for backbone EF) were phosphorylated using T4 polynucleotide kinase (T4PNK) (incubation for 30 min at 37 ^{o}C and deactivation for 10 min at 65 ^{o}C). Prior to incubation, the concentration of each sequence was adjusted to 10 µM, in the presence of 1xT4PNK buffer, 2.5 mM ATP, and 0.08 U.µL^{-1} of T4PNK. After 5' end phosphorylation, equivolumes of each of the complementary strands (A1/A2, B1/B2, A1P/A2P, B1P/B2P, C1/C2, D1/D2, C1P/C2P, D1P/D2P, E1/E2, F1/F2, E1P/E2P and F1P/F2P) were mixed and

incubated for 10 min at room temperature. An equal volume of 2x Quick ligase buffer was then added to a final concentration of 1x. Temporal growth was performed by a stepwise mixing of equal amount of duplex stocks as follows:

1. For backbone with AB repeating domains: a) A1P-A2P + B1-B2 + Quick ligase + A1-A2 + B1-B2 +A1-A2, b) B1P-B2P + B1-B2 + Quick ligase + A1-A2 +B1-B2.
2. For backbone with CD repeating domains: a) C1P-C2P + D1-D2 + Quick ligase + C1-C2, b) D1P-D2P + D1-D2 + Quick ligase.
3. For backbone with EF repeating domains: a) E1P-E2P + F1-F2 + Quick ligase + E1-E2 + F1-F2, b) F1P-F2P + F1-F2 + Quick ligase + E1-E2.

To allow for hybridization of the sticky ends of two consecutive building blocks and ligation, the mixture was incubated for 5 min after each addition step. Quick ligase was added, at an initial concentration of 10 $U.\mu L^{-1}$, after the addition of the second building block, and the mixture was incubated for 5 min. Next, fractions "a" and "b" were mixed and incubated for 15 min at room temperature. Fractions "a", "b" and the final mixture were loaded on a native 2% AGE (1xTAE, 100 V, 60 min) (Fig. S9) and the full-length product was imaged under SybrSafe channel, excised, and purified using QIAquick gel extraction KIT.

PCR was achieved using MyTaq™ HS Red Mix PCR KIT where the reactions were carried out in batches of 200 µL each ($[\text{ds-template}]_{final}$ = 0.10 $ng.\mu L^{-1}$, $[\text{forward primer}]_{final}$ = $[\text{reverse primer}]_{final}$ = 0.625 µM, and a final concentration of 1x MyTaq™ HS Red Mix) (Fig. S9). The mixtures were heated at 95 ºC for 1 min and followed by 30 cycles of: a) 95 ºC for 15 seconds, b) 64 ºC (optimized temperature) for 15 sec, and 3) 72 ºC for 15 sec. Then, the samples were purified by 2% denaturing AGE (1x ALK, 2 h, 55 V) which was imaged under Sybr Gold channel (1 min in distilled water, 15 min in 2xSSC, 15 min in 1xSSC and 1x Sybr Gold, and 15 min in 1xTAE). Full-length backbones were excised and purified by Freeze 'N Squeeze™ DNA Gel Extraction Spin Columns (manufacturer protocol was applied).

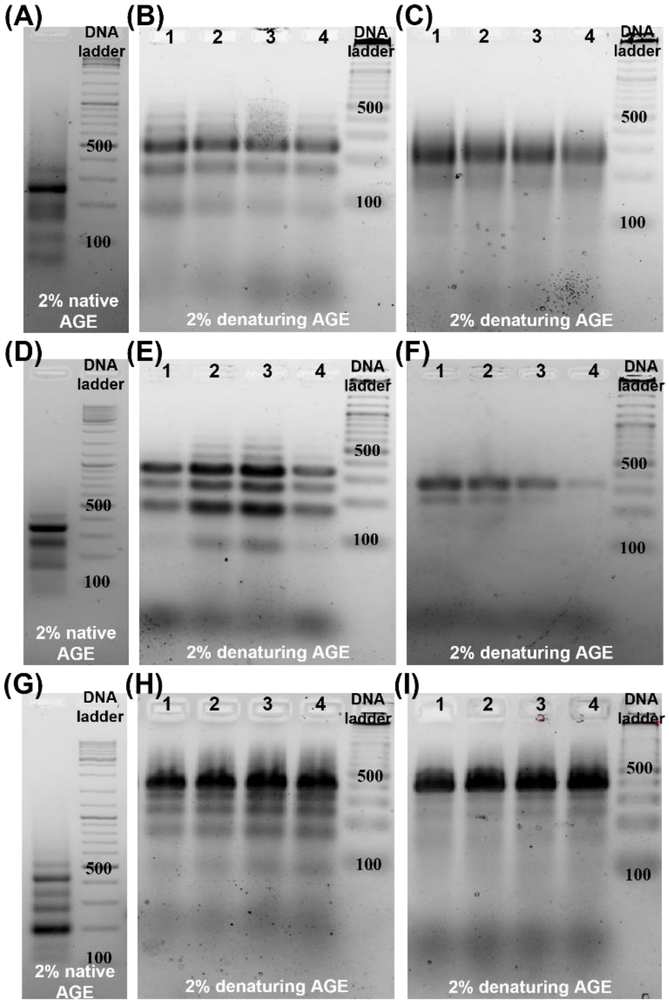

Figure S9. Temporal growth product of ds-CD6 characterized by 2% native AGE (A). PCR optimization of the isolated ds-CD6 temporal growth backbone (B). All lanes in (B) have a final template concentration of 0.10 ng.µL^{-1}. Lanes 1, 2, 3 and 4 correspond to a temperature of 61 ^{o}C, 62 ^{o}C, 63 ^{o}C and 64 ^{o}C, respectively. PCR optimization of the purified isolated ds-CD6 strand extracted after the first round of PCR (C). All lanes in (C) have a final template concentration of 0.10 ng.µL^{-1}. Lanes 1, 2, 3 and 4 correspond to a temperature of 61 ^{o}C, 62 ^{o}C, 63 ^{o}C and 64 ^{o}C, respectively.

Temporal growth product of ds-EF8 characterized by 2% native AGE (D). PCR optimization of the isolated ds-EF8 temporal growth backbone (E). All lanes in (E) have a final template concentration of 0.10 ng.µL^{-1}. Lanes 1, 2, 3 and 4 correspond to a temperature of 61 ^{o}C, 62 ^{o}C, 63 ^{o}C and 64 ^{o}C, respectively. PCR optimization of the purified isolated ds-EF8 strand extracted after the first round of PCR (F). All lanes in (F) have a final template concentration of 0.10 ng.µL^{-1}. Lanes 1, 2, 3 and 4 correspond to a temperature of 61 ^{o}C, 62 ^{o}C, 63 ^{o}C and 64 ^{o}C, respectively.

Temporal growth product of ds-AB10 characterized by 2% native AGE (G). PCR optimization of the isolated ds-AB10 temporal growth backbone (H). All lanes in (H) have a final template concentration of 0.10 ng.µL^{-1}. Lanes 1, 2, 3 and 4 correspond to a temperature of 61 ^{o}C, 62 ^{o}C, 63 ^{o}C and 64 ^{o}C, respectively. PCR optimization of the purified isolated ds-AB10 strand extracted after the first round of PCR (I). All lanes in (I) have a final template concentration of 0.10 ng.µL^{-1}. Lanes 1, 2, 3 and 4 correspond to a temperature of 61 ^{o}C, 62 ^{o}C, 63 ^{o}C and 64 ^{o}C, respectively. GeneRuler DNA ladder mix is used in all gels.

The selective extension of one arm of **T3** requires the presence of a forward primer sequence that selectively hybridizes to one temporal growth backbone (Fig. S10). Three different forward primers, matching the 3 different backbones that were generated, were added to the 3' end of the AT, DT and HT sequences resulting in AT-ABprimer (R1), DT-CDprimer (R2) and HT-EFprimer (R3).

To extend one arm, PCR was performed using one single backbone as a template (PCR conditions: the template concentration was 0.15 ng.µL^{-1} while each primer had a concentration of 0.6 µM both in 1x MyTaq™ HS Red Mix). The heating cycles are the same as described above. Same experimental conditions were used for the simultaneous elongation of 2 and 3 arms of **T3**. After each of the PCR cycles, the samples were loaded on a 2%, 1xTAE AGE. The simultaneous extension of the arms, performed under the same PCR conditions as above, resulted in the formation of sub-products corresponding to the mono-extension of individual arms and the double- extension of 2 arms.

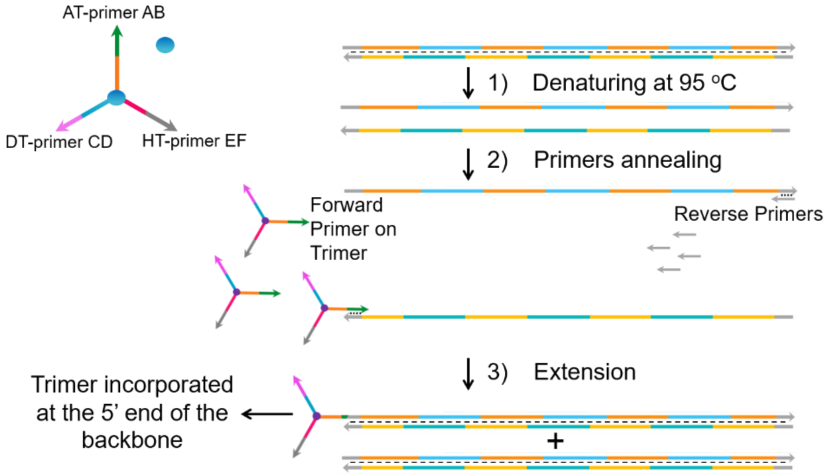

Figure S10. Schematic representation of **T3** incorporation within the backbone having repeating domains. Each arm of **T3** has a specific sequence that acts as a primer during PCR. This strategy allows the specific incorporation of temporal growth backbone within **T3**.

SI-VIII. Conversion of ds backbones and three-way DNA scaffold into ssDNA

After isolating different dsDNA backbones, conversion to ssDNA was performed using lambda exonuclease. The conversion required the use of phosphorylated reverse primers during PCR prior to nuclease treatment. Different backbones were used to assemble DX tiles with different lengths. All sequences and schematic representation of these tiles were summarized in section III.

After performing simultaneous PCR using 3 different backbones and **T3**, the elongated product that was isolated is double-stranded; therefore, its conversion into ssDNA was necessary for subsequent nanostructures' assemblies. In addition, since the 3 arms are covalently attached through the small molecule core, the conversion via denaturing AGE was possible. After PCR, the product was loaded on 1xALK agarose (2 h 30 min at 55 V) (Fig. S11) along with a molecular ruler, and the product with

expected length (1071 bps) was excised and purified using freeze and squeeze, and nucleotide cleaning KIT.

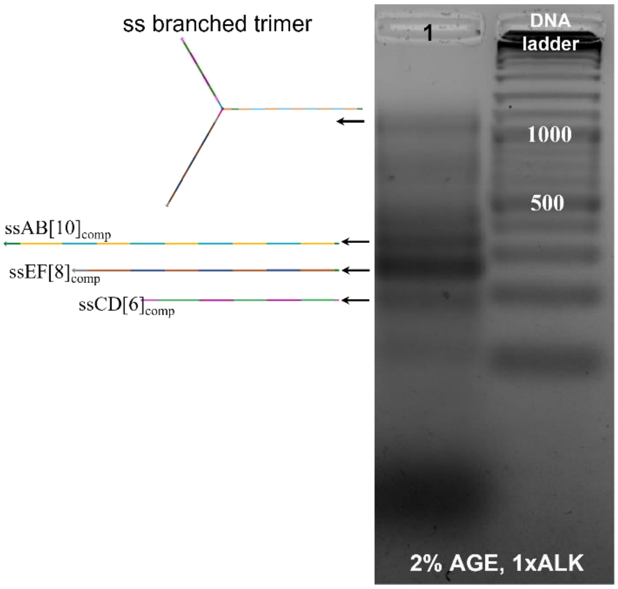

Figure S11. Single-stranded three-way DNA scaffold isolation by denaturing 2% AGE (1xALK, 55 V, 2 h 30 min). GeneRuler DNA ladder mix is used.

SI-IX. DX tile assemblies

DX tiles were assembled by annealing DX staple strands with ssDNA backbones. DX staples were added in different equivalents for different backbones; for example, DX-AB was assembled by adding 5 equivalents of A staples and 4 equivalents of B staples in 1xTAMg, and the mixture was annealed from 95 ^{o}C to 4 ^{o}C for 4 hours. Similarly, DX-EF and DX-CD were assembled by adding 4 equivalents of E staples and 3 equivalents of F staples, and 3 equivalents of C staples and 2 equivalents of D staples, respectively (Fig. S12). All individual tiles were characterized by AFM revealing the formation of rigid 1D structures (Fig. S13). Similarly, DX tiles were assembled on either each arm of the three-way DNA scaffold or on 2 arms or on all arms, and were characterized by native 1xTAE, AGE (100 V, 1 h). Contour length measurements were performed using photoshop (Fig. S14).

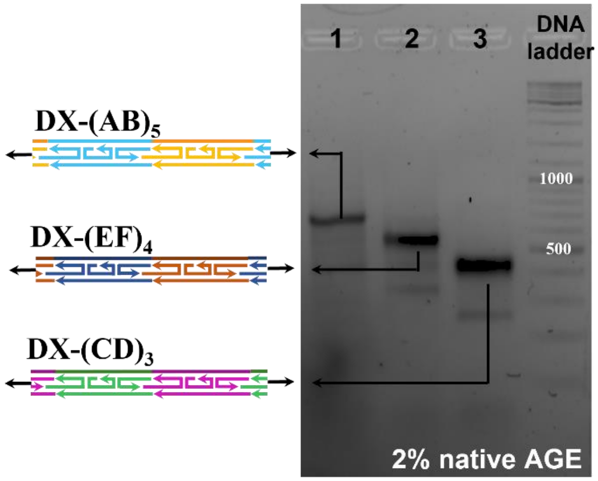

Figure S12. Native AGE (2%, 1xTAE, 100 V, 1h) characterization of DX tiles having different sizes. DX-$(AB)_5$, DX-$(EF)_4$ and DX-$(CD)_3$ were loaded in lanes 1, 2 and 3, respectively. GeneRuler DNA ladder mix is used.

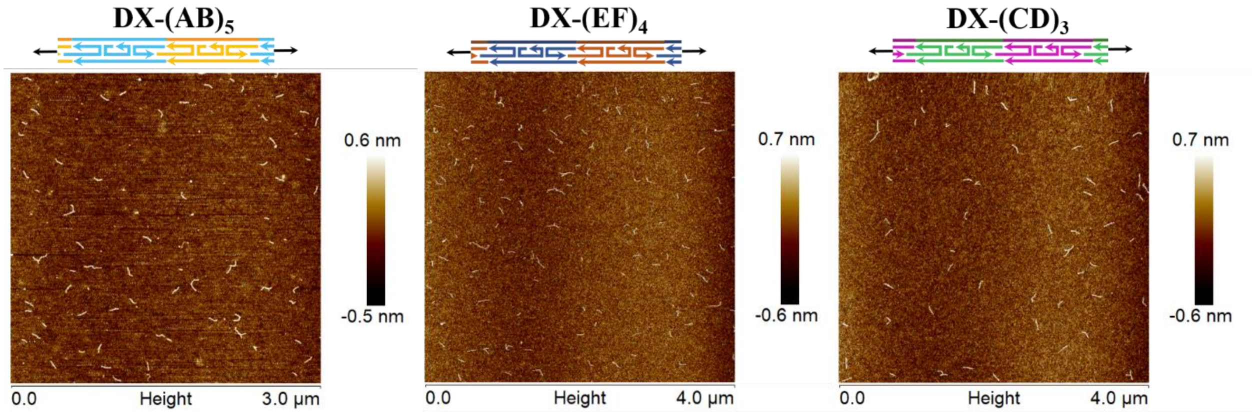

Figure S13. AFM characterization of DX tiles having different sizes.

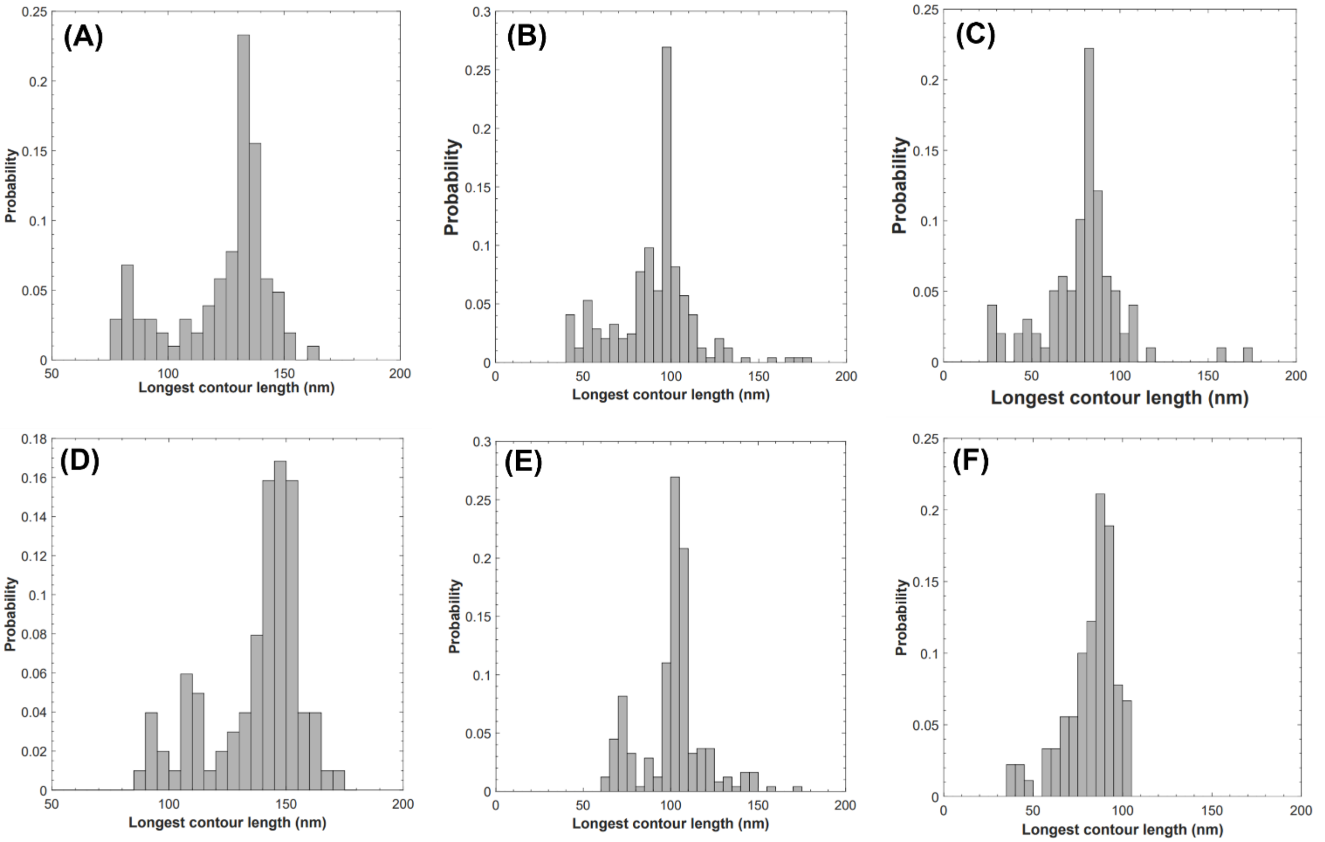

Figure S14. Histograms of measured contour lengths from AFM images of individual **DX-(AB)$_5$** (counts = 103) (A), **DX-(EF)$_4$** (counts = 245) (B), and **DX-(CD)$_3$** (counts = 100) (C). Histograms of measured contour lengths from AFM images of three-way DNA nanostructure arms **DX-(AB)$_5$** (counts = 115) (D), **DX-(EF)$_4$** (counts = 140) (E), and **DX-(CD)$_3$** (counts = 132) (F).

SI-X. Automated angle counting

A FIJI code was modified to perform an automated counting of the angles and to calculate the contour length of the structures. This code consisted, in its first step of performing thresholding, smoothing and skeletonization of AFM images (Fig. S15). The skeletons were then analyzed to locate the center of the three-way DNA nanostructure and to automatically calculate the angles between each of the arms. To do so, a vector, with a determined length was created, and started from the center of the structure pointing outwards. Mis-formed structures with insufficient arms or overlapping structures were automatically filtered to obtain a higher quality data set. The histogram of the measured angles was fitted to a gaussian distribution. From this gaussian, the mean (μ) and the standard deviation (σ) were compared for each set of three-way DNA nanostructures.

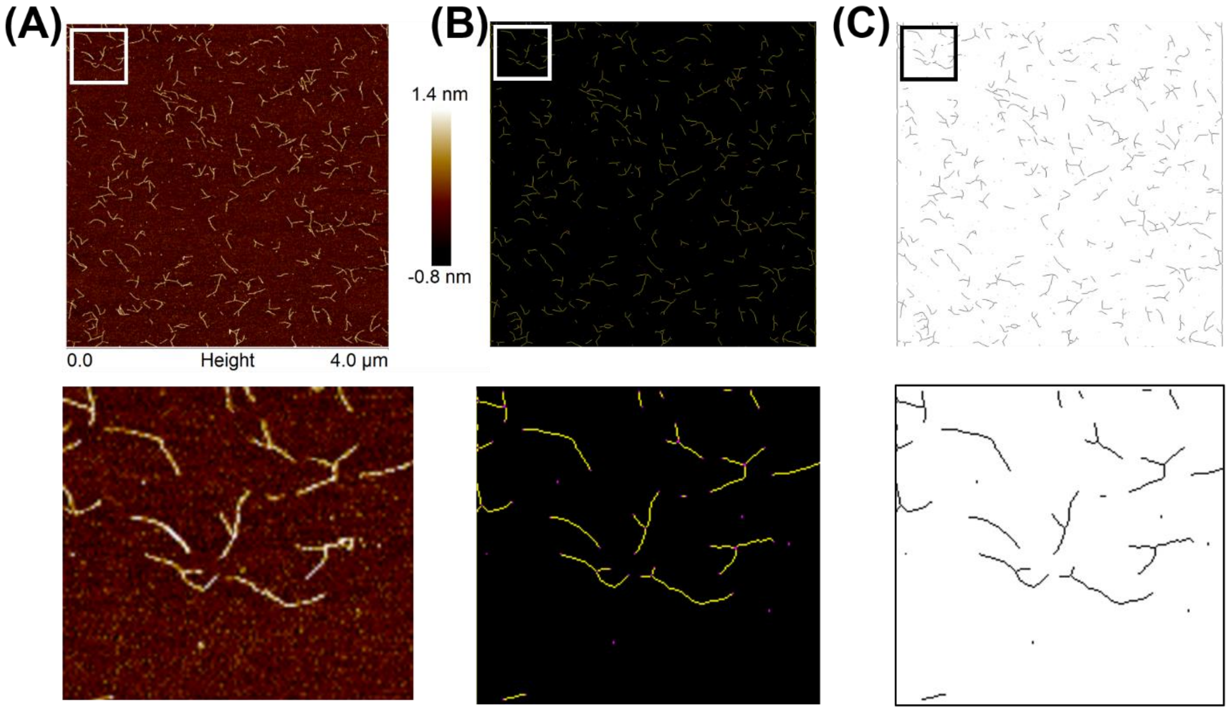

Figure S15. Conversion of an AFM image (A) featuring three-way DNA nanostructures to a skeletonized image (B and C) for subsequent automated angle counting. (Check section X for code details)

SI-XI. Nanotweezer formation and folding in response to streptavidin

DX tiles were assembled by annealing DX staple strands with single-stranded three-way DNA scaffold. To each one equivalent of scaffold, five equivalents of DX-A staples, four equivalents of DX-B staples, four equivalents of DX-E staples, three equivalents of DX-F staples, three equivalents of DX-C staples, two equivalents of DX-D staples, and five equivalents of DX-core staples were added in 1xTAMg, and the mixture was annealed from 95 ^oC to 4 ^oC for 4 hours.

Folding the three arms of the nanotweezer required the addition of one biotinylated sequence in each DX staple set (DX-A, DX-E, and DX-C). In addition, folding of two arms (**DX-EF** and **DX-CD**) required the addition of DX-E and DX-C staples with one biotinylated sequence in each set.

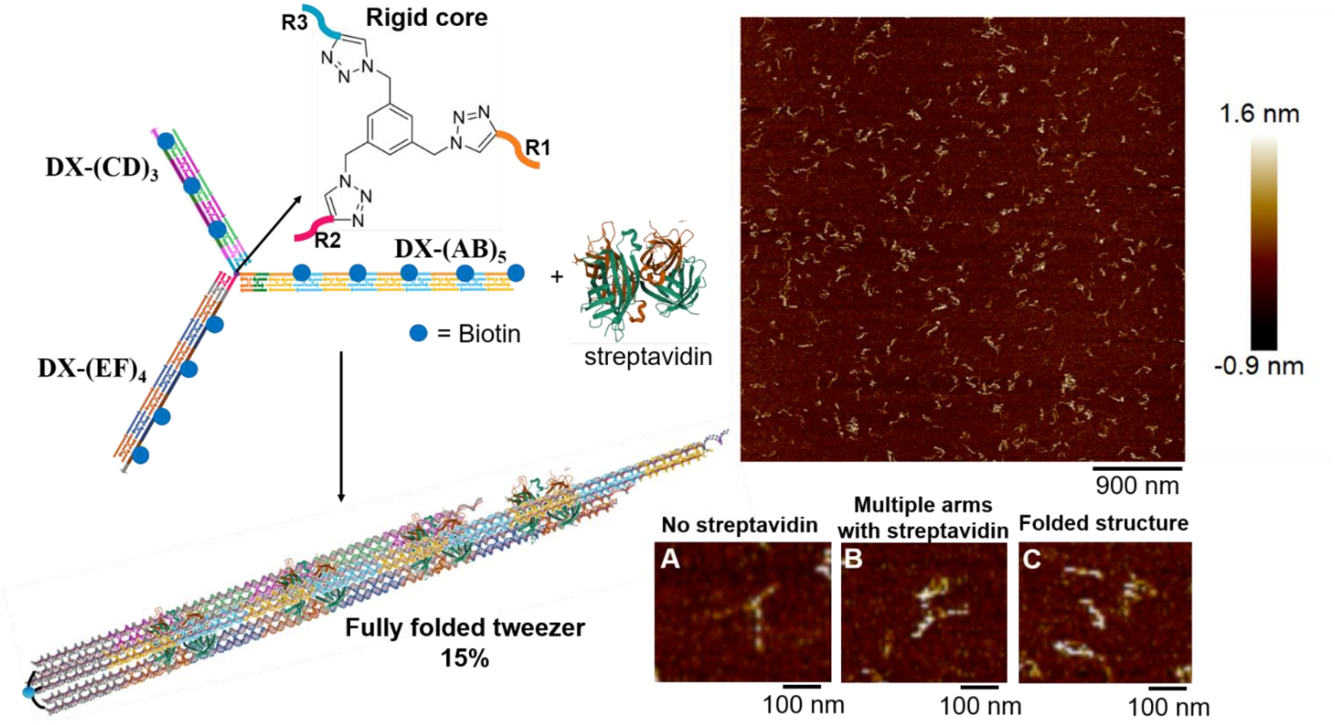

Figure S16. Schematic representation of three-way DNA nanostructure (with **rigid** core) having DX-tile structures on all arms and periodically patterned with biotinylated functionalities on all arms and incubated with streptavidin. Three different populations are detected by AFM, multiple arms with no streptavidin (A), multiple arms with streptavidin (B), and the fully folded structure corresponding to the expected linear/tubular structure (C).

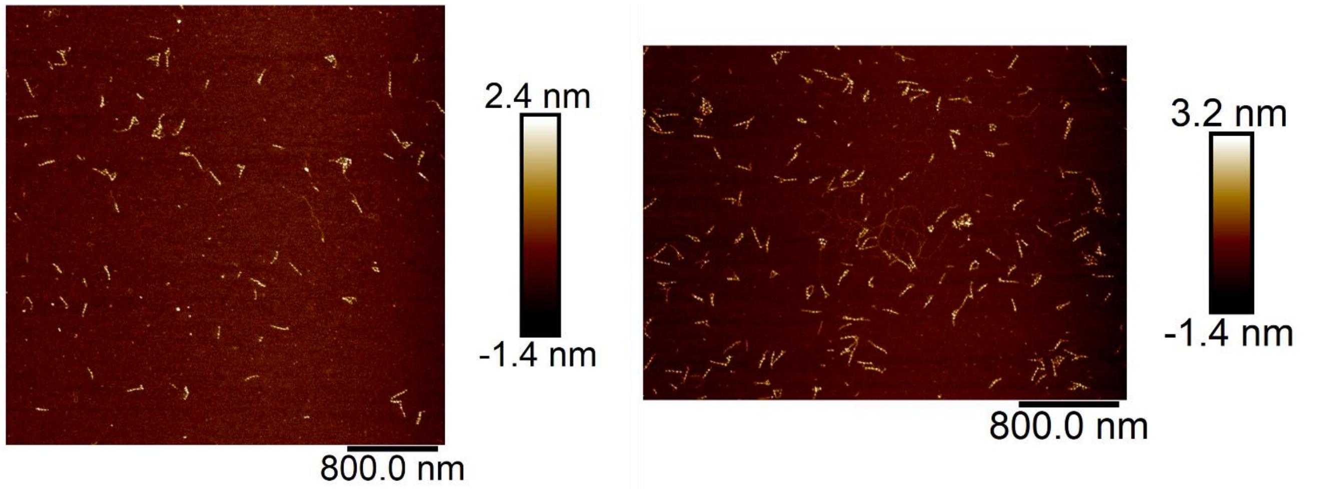

Figure S17. AFM images of the tweezer folding. Folding of all arms, featuring periodic biotin moieties, in the presence of streptavidin into a tubular-like structure.

SI-XII. Angle distribution of three-way DNA nanostructures with rigid and flexible core

We examined the angle distribution of three-way DNA nanostructures with rigid and flexible cores. The annealing mixture of DX staple strands that rigidifies all arms and the core with the input template was examined by AFM (Fig. S18) and the angles were measured in an automated fashion.

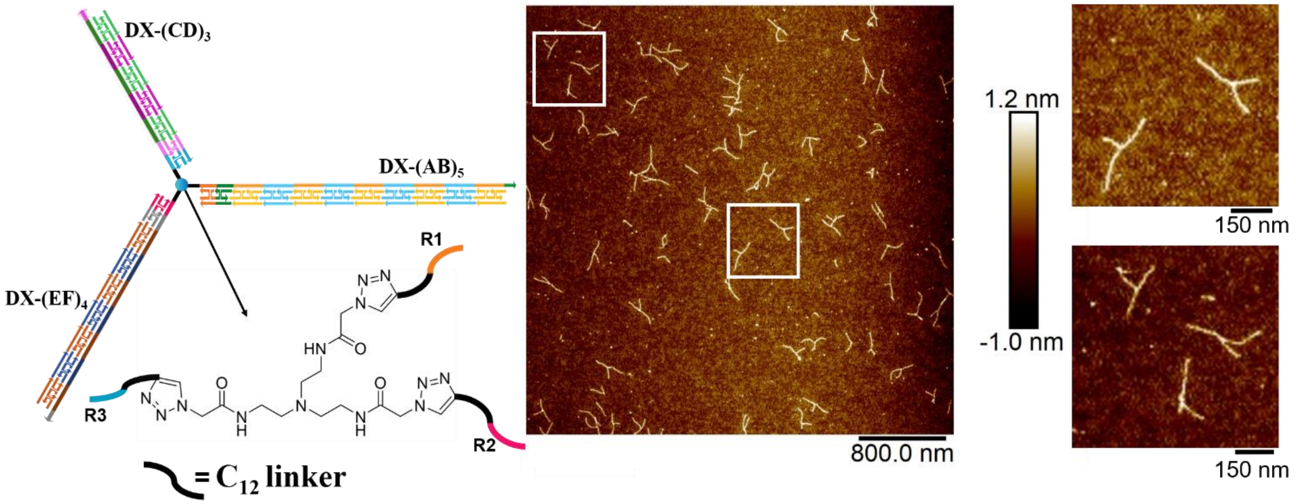

Figure S18. AFM images of three-way DNA nanostructure having elongated arms featuring DX tiles. The core of the structure consists of a flexible small molecule. The distance 'x' between the ends of **DX-AB** and **DX-EF** arms is calculated using the following equation: $x^2 = d(DX\text{-}AB)^2 + d(DX\text{-}EF)^2 - 2.d(DX\text{-}AB).d(DX\text{-}EF).\cos(120^o)$, where d(DX-AB) and d(DX-EF) are the lengths of **DX-AB** and **DX-EF** that are equal to 143 nm and 116 nm, respectively.

Going from an assembly with rigid core into an assembly with flexible core revealed a slight difference within the angle distribution where **DX-T3 (rigid)** showed an angle of $120.0^o \pm 28.7^o$ while **f-T3 (flexible)** revealed an angle of $120.0^o \pm 31.0^o$ (Fig. S19). The triazide tertiary amine core provided more flexibility than the benzene core as it possesses multiple (sp3) carbons that play a main role in the structure's flexibility; an additional C12 chain between the tertiary amine core and the DNA strand is expected to provide further flexibility.

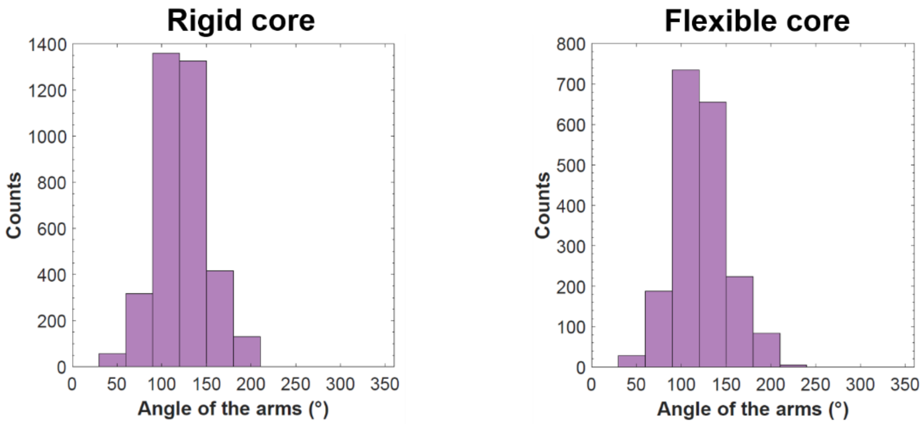

Figure S19. Histogram of angle distribution obtained by automated counting for the three-way DNA nanostructures with rigid arms having a rigid core (**DX-T3 (rigid)**) and a flexible core (**f-T3 (flexible)).**

Table S4. Mean (μ) and standard deviation (σ) values calculated from the Gaussian distribution for different types of three-way DNA nanostructures. Number of angles represents the total number of structures that contributed to the mean and standard deviation values.

Type	μ (˚)	σ (˚)	$95^\circ \leq \theta \leq 155^\circ$	Nb of angles
Rigid core	120 ± 1	28.7 ± 0.7	77.4 %	3606
Flexible core	120 ± 1	31.0 ± 2.0	75.0 %	1917

SI-XIII. AFM microscopy

AFM imaging was performed under ambient air conditions using a MultiMode8™ SPM connected to a Nanoscope™ controller from Veeco. Images were acquired using tapping mode with ScanAsyst-Air triangular silicon nitride probe (k = 0.4 N/m, tip radius = 2 nm and f_o = 70 kHz; from Bruker). All samples were diluted to a concentration between 1 nM and 5 nM in 1xTAMg. 4 μL of sample was deposited into a freshly cleaved mica surface and incubated for 30 seconds at room temperature to allow DNA adsorption, followed by 3 rounds of washing each with 50 μL of filtered milliQ water (0.22 μm Millipore filters). After last round of washing, excess liquid was removed with a strong flow of air and the samples were dried under vacuum for at least 30 min prior to imaging. Images were analyzed using NanoScope Analysis 1.40 Software and flattening was used to correct tilt, bow and scanner drift.

References:

1. T. Trinh, D. Saliba, C. Liao, D. de Rochambeau, A. L. Prinzen, J. Li and H. F. Sleiman, *Angewandte Chemie*, 2019, **131**, 3074-3079.
2. G. D. Hamblin, J. F. Rahbani and H. F. Sleiman, *Nature communications*, 2015, **6**, 7065.
